# Factors associated with diet quality of adolescents in Saudi Arabia

**DOI:** 10.3389/fpubh.2024.1409105

**Published:** 2024-08-21

**Authors:** Walaa Abdullah Mumena

**Affiliations:** ^1^Clinical Nutrition Department, College of Applied Medical Sciences, Taibah University, Madinah, Saudi Arabia; ^2^Saudi Electronic University, Riyadh, Saudi Arabia

**Keywords:** diet quality, factors, associations, adolescents, Saudi Arabia

## Abstract

**Introduction:**

Research exploring factors that may influence the diet quality of adolescents in the Middle East are very limited. We aimed to investigate factors associated with diet quality and the weight status of adolescents in Saudi Arabia.

**Methods:**

A cross-sectional study that included 638 healthy adolescents aged between 11 and 18 years who were randomly recruited from 16 private and public middle- and high-schools located in two Saudi cities (Jeddah and Madinah). All participants were given an envelope for parents to collect socioeconomic data. Diet quality and anthropometric data of adolescents were evaluated at school.

**Results:**

Median diet quality score was higher among males compared to female adolescents (10.00 (8.00–11.00) vs. 9.00 (8.00–10.0), respectively, *p* = 0.018). Median diet quality score was significantly higher among adolescents residing in Jeddah compared to adolescents residing in Madinah (10.00 (9.00–11.0) vs. 9.00 (8.00–10.0), respectively, *p* = 0.002). Stepwise linear regression analysis indicated that city of residence (B = −0.53, SE = 0.16 [95% CI: −0.83 to −0.22]), and child’s sex (B = −0.34, SE = 0.15 [95% CI: −0.64 to −0.05]) were associated with diet quality scores of adolescents in Saudi Arabia.

**Discussion:**

Future longitudinal research should be directed to further investigate other possible factors influencing the diet quality of adolescents and individuals from other age groups in Saudi Arabia.

## Introduction

Diet quality is an indicator of the variety of the key food groups relative to the dietary guideline recommendations. It involves the assessment of not only the variety of food but also the quality of the diet. Diet quality allows the examination of the epidemiological associations between foods and health (1–3). Diet quality is found to be inversely associated with the risk of several health outcomes in children, adolescents, and adults (1, 3). For example, diet quality has been linked to weight status, metabolic syndrome, blood pressure, cognitive performance, and quality of life (3).

Different methods have been used to assess the diet quality of individuals, each of which use different scoring system that represent both quality and variety of diets. Indicators mainly fall within one of the three categories: (1) Food/food group indicators; (2) Nutrient-base indicators; (3) Combined indices (2, 3). The use of scoring system that consider both food group and nutrient indicators has become more popular. Recently, the Short-Form Food Frequency Questionnaire (SFFFQ) has been used repeatedly used among healthy and ill individuals (4–6).

Several factors may influence the diet quality of young individuals. Gender inequality has been shown to have an impact on various nutrition-related aspects (7, 8). Socioeconomic factors, including maternal education, occupation, and income, have been also found to influence the diet quality of adolescents (9, 10). Data obtained from a large study conducted among children and caregivers in Ireland suggest that household socioeconomic factors (e.g., maternal education level, household social class, household income) are important factors that can be associated with the dietary quality of children (11). Educated mothers are more likely to have healthy food options at home (e.g., fruits and vegetables) (12). Additionally, it was found that the children of full-time working mothers had more unhealthy eating behaviors and food choices (13, 14). Family income is also a crucial factor that may affect the diet quality of young individuals, as it reflects the availability and accessibility of nutritious food (12, 15, 16).

Negative dietary behaviors such as skipping meals and fast-food consumption are linked to lower diet quality (17–21). In addition, data among young adults in Saudi Arabia have shown low consumption of fruits, vegetables, nuts, and fish, while high consumption of processed foods and sugar-sweetened beverages have been reported (22). These unhealthy dietary behaviors are common among adolescents in Saudi Arabia and in other populations (17, 18, 23–25). However, limited research has investigated factors associated with diet quality and the weight status of adolescents in Saudi Arabia. Thus, this study aimed to explore factors associated with diet quality of adolescents in Saudi Arabia. Such data are needed to plan effective interventions that target improving the diet quality of adolescents in Saudi Arabia and in other similar settings in the Middle East.

## Materials and methods

### Study population and sampling

For this cross-sectional study, adolescents aged between 11 and 18 years were recruited from 16 schools located in two major cities in the Western region of Saudi Arabia, Jeddah and Madinah. Schools were chosen at random from different areas in both cities (North, South, East, West) which includes middle and high schools, boys’ and girls’ schools, private and public schools, and schools located at relatively wealthy neighborhoods and schools from lower income communities. Exclusion criteria include chronic diseases, allergies, diet restrictions, and use of medications that may affect weight status. The minimum sample size needed for this was estimated to be 235 adolescents based on beta = 0.10, alpha = 0.05 (two sided), mean dietary quality score of 11.4, a detectable difference of 4% in mean value between male and female adolescents, and a standard deviation of 1.6 (4, 26). Ethical certificate for this study was granted from the ethical review board in the College of Applied Medical Sciences, Taibah University (2021/104/201 CLN). Signed consent form was obtained from caregivers of all adolescences before collecting the data.

### Data collection

**Figure 1:**
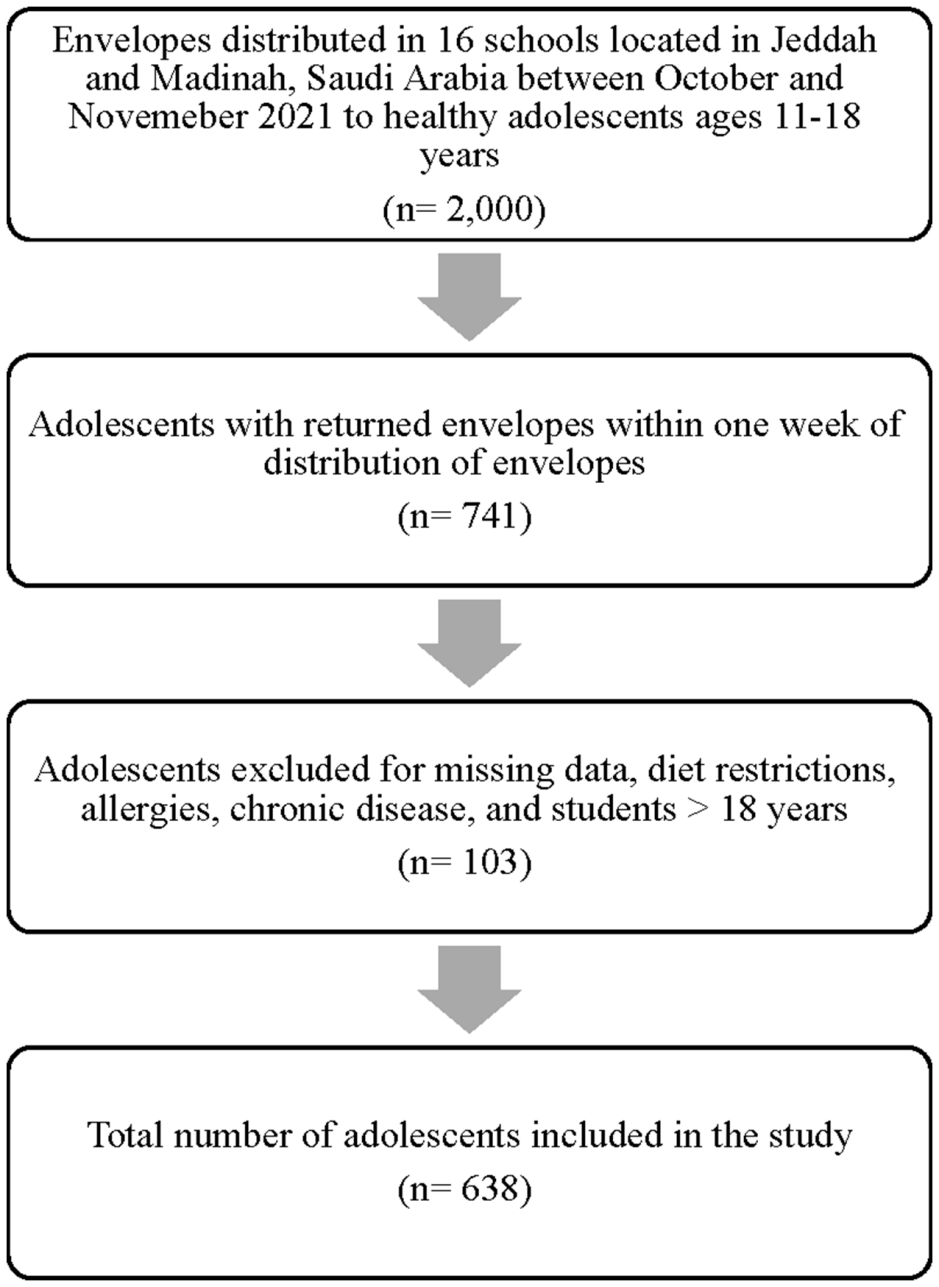
Flowchart of study sample.

One hundred twenty-five envelopes were distributed by a trained data collection team members to students in each school with a total of 2,000 envelopes (Figure 1). Each envelope included a consent form to be signed by the student’s mother in addition to a questionnaire to collect data on sociodemographic/socioeconomic status and height and weight of mothers. Mothers’ body mass index (BMI) was later calculated and categorized based on the World Health Organization (WHO) criteria (underweight, healthy weight, overweight, and obesity) (27). The envelopes were distributed to students between October and November 2021 in random classes in each school and data collection team members collected all returned envelopes within a maximum of 1 week from the date of distribution. Dietary data and anthropometrics of adolescents were then collected at school from each student.

### Assessment of diet quality

Diet quality was evaluated using the SFFFQ, which is a modified version of the original tool that was developed by Cleghorn et al. (4). Two nutritionists with expertise in dietary assessment validated items included in the SFFFQ to ensure the inclusion of all items consumed by adolescents in Saudi Arabia. A hard copy of the SFFFQ was filled out by each student during the school day; Instructions concerning the frequency of consumption and serving size were delivered by trained data collection personnel. The SFFFQ includes 20 food items as follows: (1) “Fruits (tinned/fresh/frozen)”; (2) “Fruit juices (not including syrup), fruit drink (e.g., Vimto) or powder juice (e.g., Tang)”; (3) “Salad (not including garnish added to sandwiches)”; (4) “Cooked vegetables (not including potatoes)”; (5) “Fried potatoes/chips”; (6) “Beans or legumes (baked beans, chickpeas, lentils)”; (7) “Fiber-rich breakfast cereal (e.g., Fit-ness)”; (8) “Whole wheat bread (all kinds including rusks)”; (9) “Cheese/yogurt”; (10) “Crisps/savory snacks”; (11) “Sweet biscuits, cookies, cakes, chocolates, and sweets)”; (12) “Ice cream/cream (cooking cream or whipped cream or cream)”; (13) “Soft drinks/pop/iced tea/energy drinks/beer (not sugar free or diet/light)”; (14) “Beef or lamb (steak, mince, cube)”; (15) “Chicken or turkey (steak, mince, cube) does not include those in batter or with added breadcrumbs”; (16) “Processed meats/meat product (sausage, bacon, meat samosa, corned beef, meat pies/pastries, meat burgers)”; (17) “Processed chicken/turkey (chicken nuggets/burgers, chicken pies in batter or bread-crumbs)”; (18) “White fish in batter or breadcrumbs (fish fingers and fried fish)”; (19) “White fish (does not include fish in batter or breadcrumbs)”; (20) “Oily fish (salmon, sardines, fresh tuna-not tinned tuna.” The frequency of consumption for items 1–13 were: “rarely or never” (coded as 0); “less than once a week” (coded as 1); “once a week” (coded as 2); “2–3 times a week” (coded as 3); “4–6 times a week” (coded as 4); “1–2 times a day” (coded as 5); “3–4 times a day” (coded as 6); “≥ 5 a day” (coded as 7). For items 14–20, the frequencies of consumption were: “rarely or never” (coded as 0); “less than once a week” (coded as 1); “once a week” (coded as 2); “2–3 times a week” (coded as 3); “4–6 times a week” (coded as 4); “≥7 times a week” (coded as 5). The excel sheet provided by Nutritools (https://www.nutritools.org) was used to calculate the total score of diet quality which ranges between 5 and 15.

### Assessment of anthropometrics

After each student handed in the completed SFFFQ, students were directed to move to the anthropometric data collection station. The data collection personnel measured the height and weight of all students following a standardized procedure (28). Height was measured in centimeters by a measuring tape that was assembled on a flat wall. Students were requested to take off their shoes and look straight forward to assess their height and recoded the number after rounding it to the nearest 0.5 centimeter. An electronic scale (OMRON BF508, Japan) was used to assess weight of students in kilograms, and the measurement was rounded to the nearest 0.1 kilogram. Measurements of height and weight were collected twice and average for each student was calculated using Excel. Third measurement was collected if the height and weight recorded in the first two times have difference of more than 0.5 centimeter and 0.01 kilogram. Height, weight, and date of birth were used to determine the weight status of students using body mass index percentile according to the criteria of the Center for Disease Control and Prevention (CDC) (29).

### Statistical analysis

Data were analyzed using the Statistical Package for the Social Sciences (SPSS 20, SPSS Inc., Chicago, IL). Data of categorical variables are presented as frequency (percentage), while data of continuous variables are presented as mean ± standard deviation and median (interquartile range). Normality of the diet quality score distribution was evaluated using the Shapiro–Wilk test. Mann–Whitney U test or Kruskal-Wallis H test were used to compare the median of diet quality score across the different groups. Pairwise comparison was performed to explore the significance across the different income groups. Bonferroni adjustments were used to correct for multiple testing in the bivariate analysis. Stepwise regression analysis was performed to explore factors (independent variables) associated with diet quality score (dependent variable) among the study participants. All tests were two-tailed with alpha = 0.05.

## Results

### Sample characteristics

A total of 741 envelopes were returned. Data of 638 adolescents were included in this study after excluding 6.48% (*n* = 48) of the adolescents due to missing dietary or anthropometric data, 4.59% (*n* = 34) for food allergy, 2.16% (*n* = 16) for chronic diseases, 0.40% (*n* = 3) of the adolescents were excluded as they were on a diet regimen, and 0.27% (*n* = 2) of the adolescents were excluded as they were older than 18 years. Fifty-three percent (*n* = 339) of adolescents aged between 15 and 18 years and 52.8% of the adolescents were female (*n* = 337). Most of the adolescents were Saudis (82.4%, *n* = 526), and 64.6% of participants (*n* = 412) were from Madinah. Fifty-six percent (*n* = 359) of adolescents were enrolled in public schools, while 46.1% (*n* = 294) of adolescents were the middle children. The prevalence of underweight (<5th percentile) among adolescents included in the study was 9.25% (*n* = 59), whereas the prevalence of overweight and obesity (≥85% percentile) was 36.5% (*n* = 233). About half of the mothers included in this study aged 41–50 years (44.7%, *n* = 285), and 91.2% (*n* = 582) were married. Over half of the mothers (53.4%, *n* = 310) were experiencing overweight or obesity (≥25 km/m^2^), and 61.0% (*n* = 389) reported holding a Bachelor or Postgraduate degree. Over two-thirds of the mothers (34.6%, *n* = 221) were employed of which 55.2% (*n* = 122) mothers were working 7–8 h per day. Twenty-two percent of the mothers (*n* = 140) reported family monthly income of < SR 6,000 (see Table 1).

**Table 1:** Characteristics of the study sample (*n* = 638).

	*n*	%
Adolescents		
School level		
Middle school	318	49.8
High-school	320	50.2
Age group		
11–14 years	299	46.9
15–18 years	339	53.1
Sex		
Males	301	47.2
Females	337	52.8
Nationality		
Saudi	526	82.4
Non-Saudi	112	17.6
City of residence		
Madinah	412	64.6
Jeddah	226	35.4
School type		
Private	279	43.7
Public	359	56.3
Order of child among siblings		
Older child	177	27.7
Middle child	294	46.1
Youngest child	154	24.1
Only child	13	2.04
Weight status		
Underweight (<5th percentile)	59	9.25
Healthy weight (5th to <85th percentile)	346	54.2
Overweight (85th to <95th percentile)	102	16.0
Obesity (≥95th percentile)	131	20.5
Mothers		
Age group		
19–40 years	278	43.6
41–50 years	285	44.7
>50 years	75	11.8
Marital status		
Married	582	91.2
Separated	38	5.96
Widow	18	2.82
Weight status^1^		
Underweight (<18.5 km/m^2^)	16	2.76
Healthy weight (18.5–24.9 km/m^2^)	254	43.8
Overweight (25.0–29.9 km/m^2^)	192	33.1
Obesity (≥30 km/m^2^)	118	20.3
Education level		
≤High school/Diploma	249	39.0
Bachelor degree	324	50.8
Postgraduate degree	65	10.2
Employment status		
Unemployed//Retired	417	65.4
Employed	221	34.6
Working hours per day		
<4 h	5	0.78
4–6 h	72	11.3
7–8 h	122	19.1
>8 h	22	3.45
N/A	417	65.4
Family monthly income in SR^2^		
<SR 6,000	140	21.9
SR 6,000–10,999	154	24.1
SR 11,000–15,999	120	18.8
SR 16,000–20,999	110	17.2
≥SR 21,000	114	17.9

1 Data concerning weight status for mother were provided by 580 mothers.

2 SR: Saudi Riyal ($1 = SR 3.75).

### Association between diet quality and characteristics of adolescents

Diet quality score reported among the study sample ranged between 5 and 14. Median diet quality score was significantly higher among the adolescents residing in Jeddah compared to the adolescents residing in Madinah (10.00 (9.00–11.0) vs. 9.00 (8.00–10.0), respectively, *p* = 0.002). Additionally, median diet quality score was significantly higher among male compared to female adolescents (10.00 (8.00–11.00) vs. 9.00 (8.00–10.0), respectively, *p* = 0.018). Diet quality score was significantly linked to family monthly income but results of the pairwise comparison indicated no differences in median diet quality score across the different family income groups after Bonferroni adjustment (*p* > 0.005 for all tests). The median diet quality score was similar among all other characteristics of adolescents and their mothers included in this study (see Table 2).

**Table 2:** Median diet quality score across the different groups (*n* = 638).

	Mean ± SD	Median (IQR)	*p*-value
Adolescents			
School level			
Middle school	9.18 ± 1.78	9.00 (8.00–10.0)	0.201
High-school	9.33 ± 1.68	9.00 (9.00–10.0)	
Age group			
11–14 years	9.17 ± 1.76	9.00 (8.00–10.0)	0.116
15–18 years	9.33 ± 1.70	9.00 (9.00–11.0)	
Sex			
Males	9.43 ± 1.73	10.00 (8.00–11.00)	**0.018** ^1^
Females	9.10 ± 1.71	9.00 (8.00–10.0)	
Nationality			
Saudi	9.21 ± 1.76	9.00 (8.00–10.0)	0.235
Non-Saudi	9.46 ± 1.54	9.50 (9.00–10.0)	
City of residence			
Madinah	9.08 ± 1.75	9.00 (8.00–10.0)	**0.002** ^1^
Jeddah	9.56 ± 1.65	10.00 (9.00–11.0)	
School type			
Private	9.29 ± 1.77	9.00 (8.00–11.0)	0.795
Public	9.23 ± 1.70	9.00 (8.00–10.0)	
Order of child among siblings			
Older child	9.45 ± 1.80	10.00 (8.00–11.0)	0.370
Middle child	9.13 ± 1.78	9.00 (8.00–10.0)	
Youngest child	9.27 ± 1.55	9.00 (8.00–10.0)	
Only child	9.23 ± 1.48	10.00 (7.50–10.0)	
Weight status			
Underweight (<5th percentile)	9.05 ± 1.80	9.00 (8.00–10.0)	0.546
Healthy weight (5th to <85th percentile)	9.21 ± 1.73	9.00 (8.00–10.0)	
Overweight (85th to <95th percentile)	9.35 ± 1.82	9.50 (8.00–11.0)	
Obesity (≥95th percentile)	9.38 ± 1.63	9.00 (9.00–10.0)	
Mothers			
Age group			
19–40 years	9.22 ± 1.81	9.00 (8.00–11.0)	0.888
41–50 years	9.26 ± 1.70	9.00 (8.00–10.0)	
>50 years	9.33 ± 1.53	9.00 (9.00–11.0)	
Marital status			
Married	9.23 ± 1.72	9.00 (8.00–10.0)	0.286
Separated	9.58 ± 1.70	10.00 (9.00–10.25)	
Widow	9.44 ± 1.98	10.00 (9.00–11.0)	
Weight status^2^			
Underweight (BMI <18.5 kg/m^2^)	9.38 ± 1.67	9.00 (8.25–10.0)	0.800
Healthy weight (BMI 18.5 to 24.9 kg/m^2^)	9.26 ± 1.77	9.00 (8.00–10.25)	
Overweight (BMI 25.0 to 29.9 kg/m^2^)	9.13 ± 1.83	9.00 (8.00–10.00)	
Obesity (≥30.0 kg/m^2^)	9.34 ± 1.58	9.00 (9.00–10.0)	
Education level			
≤High school/Diploma	9.22 ± 1.77	9.00 (8.00–10.0)	0.051
Bachelor’s degree	9.18 ± 1.72	9.00 (8.00–10.0)	
Postgraduate degree	9.74 ± 1.59	10.00 (9.00–11.0)	
Employment status			
Unemployed/Retired	9.27 ± 1.71	9.00 (8.00–10.0)	0.611
Employed	9.22 ± 1.76	9.00 (8.00–10.0)	
Working hours per day			
<4 h	9.00 ± 1.00	9.00 (8.00–10.0)	0.797
4–6 h	9.25 ± 1.79	9.00 (8.00–10.0)	
7–8 h	9.16 ± 1.76	9.00 (8.00–10.0)	
>8 h	9.55 ± 1.68	10.00 (9.00–11.0)	
N/A	9.27 ± 1.72	9.00 (8.00–10.0)	
Family monthly income in SR^3^			
<SR 6,000	9.41 ± 1.56	10.00 (9.00–10.0)	0.025^1^
SR 6,000–10,999	9.01 ± 1.71	9.00 (8.00–10.0)	
SR 11,000–15,999	9.03 ± 1.65	9.00 (8.00–10.0)	
SR 16,000–20,999	9.29 ± 1.93	10.00 (8.00–11.0)	
≥SR 21,000	9.58 ± 1.78	10.00 (9.00–11.0)	

1 Alpha = 0.05.

2 Data concerning weight status for mothers were provided by 582 participants.

3 SR: Saudi Riyal ($1 = SR 3.75).

Stepwise linear regression analysis indicated that sex city of residence (B = −0.53, SE = 0.16 [95% CI: −0.83 to −0.22], *p* = 0.001), and child’s sex (B = −0.34, SE = 0.15 [95% CI: −0.64 to −0.05], *p* = 0.023) were associated with diet quality scores of adolescents in Saudi Arabia. School level, age group, nationality, school type, order of child among siblings, weight status of adolescents as well as age group of mothers, marital status of mothers, weight status of mothers, education level of mothers, employment status of mothers, working hours per day, and family monthly income were not associated with the diet quality of adolescents (see Table 3).

**Table 3:** Stepwise regression analysis of association between diet quality and characteristics of the study sample (*n* = 638).

Variables	Beta	Standard error	95% confidence interval	*p*-value	R-square
Model 1					
City of residence	−0.58	0.16	−0.89 to −0.28	<0.001^1^	0.03
Model 2					
City of residence	−0.53	0.16	−0.83 to −0.22	0.001^1^	0.04
Child’s sex	−0.34	0.15	−0.64 to −0.05	0.023^1^	

^1^Alpha = 0.05.

## Discussion

This study aimed to investigate factors associated with diet quality of adolescents in Saudi Arabia. Bivariate analysis shows that median diet quality score was higher among males compared to female adolescents. Additionally, median diet quality score was significantly higher among adolescents residing in Jeddah compared to adolescents residing in Madinah. Median diet quality score was similar across the different family income groups. Stepwise linear regression analysis indicated that city of residence and child’s sex were associated with diet quality scores of adolescents in Saudi Arabia.

We found that male adolescents have significantly higher diet quality scores compared to female adolescents. This finding was consistent with previous work conducted among Canadian and Brazilian adolescents (20, 30). However, other studies conducted among adolescents in the UK and Malaysia show significantly higher diet quality scores among female adolescents compared to males (31, 32). In fact, this variation in findings could be due to differences in food preference among the studied populations. Studies that reported better diet quality among males compared to female adolescents also reported higher intake of fats and lower intake of grain products, fruits and vegetables, milk products, and meats among female adolescents (20), while in Saudi Arabia higher intake of fruits was observed among males than female adolescents (33). Additionally, female adolescents are more likely to skip meals compared to male adolescents, where male adolescents reported regularity in the consumption of breakfast, lunch, and dinner compared to female adolescents (34). Data suggest a link between skipping meals and snacking behaviors, which are also found to be different between male and female adolescents (35). A study conducted in 2016 found a high correlation between adolescents and snacking while watch television. This study also found a positive association between snacking and energy intake, consumption of sugar-sweetened beverage, and frequent fast-food intake. Negative association was reported between snacking and fruit and vegetable consumption (36). In Saudi Arabia, female adolescents are more likely to stay home, which may lead to higher media use, lower dietary practices, and a lower diet quality compared to male adolescents.

Several factors related to the environment may influence the diet quality of individuals. In this study, the diet quality score was significantly higher among adolescents residing in Jeddah compared to adolescents residing in Madinah. Access to food retails, types, and prices of food offered in these outlets can affect food choices of adolescents (37). It is possible that individuals living in bigger cities, such as Jeddah, tend to consume more fruits, vegetables, and healthy food items as bigger cities have several large food retails in most, if not all, main neighborhoods that offer variety of fresh local and imported food products throughout the year, whereas individuals residing in relevantly smaller cities, like Madinah, may have limited access to these type of food retails. In addition, more job opportunities with higher income might be more available in larger cities compared to smaller cities. More research should be directed to explore association between diet quality and city of residence considering the number of food retails and type and prices of food offered.

It has been recognized that lower income households are purchasing less healthful food items, including fruits, vegetables, and dairy products, compared with higher income households (38, 39). Thus, young individuals living in lower income households tend to consume diets that are of lower quality (11, 40). However, our findings indicate similar median diet quality score across the different family income groups. Generally, the population of Saudi Arabia, including young adults, are consuming diets that are low in nutritious food items including nuts, fruits, vegetables, fish, and high quantitates of sugary drinks and processed meats, which indicate limited quality of diet (22). Inconsistent findings reported in this study can be explained by the low variation in diet quality among adolescents included in this study. Additionally, a number of factors can influence the quality of diet of low-income individuals including knowledge related coping strategies and availability of assistant programs for low-income families (39, 41–43). Several assistant programs are offered to low-income families and individuals in Saudi Arabia, including the financial support provided via the Human Resources Development Fund, citizen account, Ehsan, charities, and Food Preservation Societies (Hefz Al Nema) and many other formal and informal forms of support (41–43). These assistant programs could help in improving the quality of diet of low-income families. Improving the income of families with children is important; however, educating mothers on how to improve the diet quality of children and how to cope with food insecurity is more important to achieve optimal nutritional health on the long-term (44).

It is important to conduct interventions that focuses on improving the quality of diet from a young age in order to enhance the overall health status of individuals. A review published in 2023 reported differences in motivational factors influencing food habits among male and female adolescents. Female adolescents were found to be more externally motivated (e.g., change dietary habits to lose weight to fit traditional norms), whereas male adolescents were found to be more internally motivated (e.g., eat for enjoyment). Thus, using sex-specific motivation approaches to improve food habits of adolescents can result in more effective interventions (45). Despite the significantly higher diet quality score reported in male adolescents compared to female adolescents included in our study (10.00 vs. 9.00, respectively), the diet quality score is considered limited for both sexes (maximum diet quality score is 15), which indicates the need for tailored intervention that aims to improve the diet quality of adolescents in Saudi Arabia.

To our knowledge, this is the first study to investigate factors associated with the diet quality among adolescents in Saudi Arabia and in the Middle East. In addition, recruitment in this study was performed randomly, which increases the generalizability of findings. However, the study is limited by its design, as causal relationship cannot be determined from cross-sectional studies. In addition, findings of this study might be limited to adolescents residing in main cities in the Western region of Saudi Arabia and results could be different in other regions in Saudi Arabia. The SFFFQ used was developed and validated among population of the UK; however, the modified version of the tool used in this study included local food items as examples of the 20 food items presented in the original tool. Additionally, the SFFFQ provides total score of diet quality and data concerning the score of each component of the diet is not available.

In conclusion, the diet quality of adolescents was found to be linked to city of residence and child’s sex. Nutrition education programs targeting female adolescents and adolescents residing in smaller cities are needed to improve the diet quality of adolescents. By better understanding how sex and setting affect the quality of diets, effective interventions can be designed to improve the diet quality of adolescents in Saudi Arabia specifically and more generally in the Middle East. Future longitudinal research should be directed to further investigate other possible factors influencing the diet quality of adolescents and individuals from other age groups in order to design effective interventions that aim to improve the quality of diets among the population of Saudi Arabia.

## Data Availability

The raw data supporting the conclusions of this article will be made available by the authors, without undue reservation.
