# Supplementary material for: Factors associated with diet quality of adolescents in Saudi Arabia
Source: Front Public Health. 2024 Aug 21;12:1409105. doi: 10.3389/fpubh.2024.1409105 (PMC11371742; doi:10.3389/fpubh.2024.1409105)

Factors Associated with Diet Quality of Adolescents in Saudi Arabia

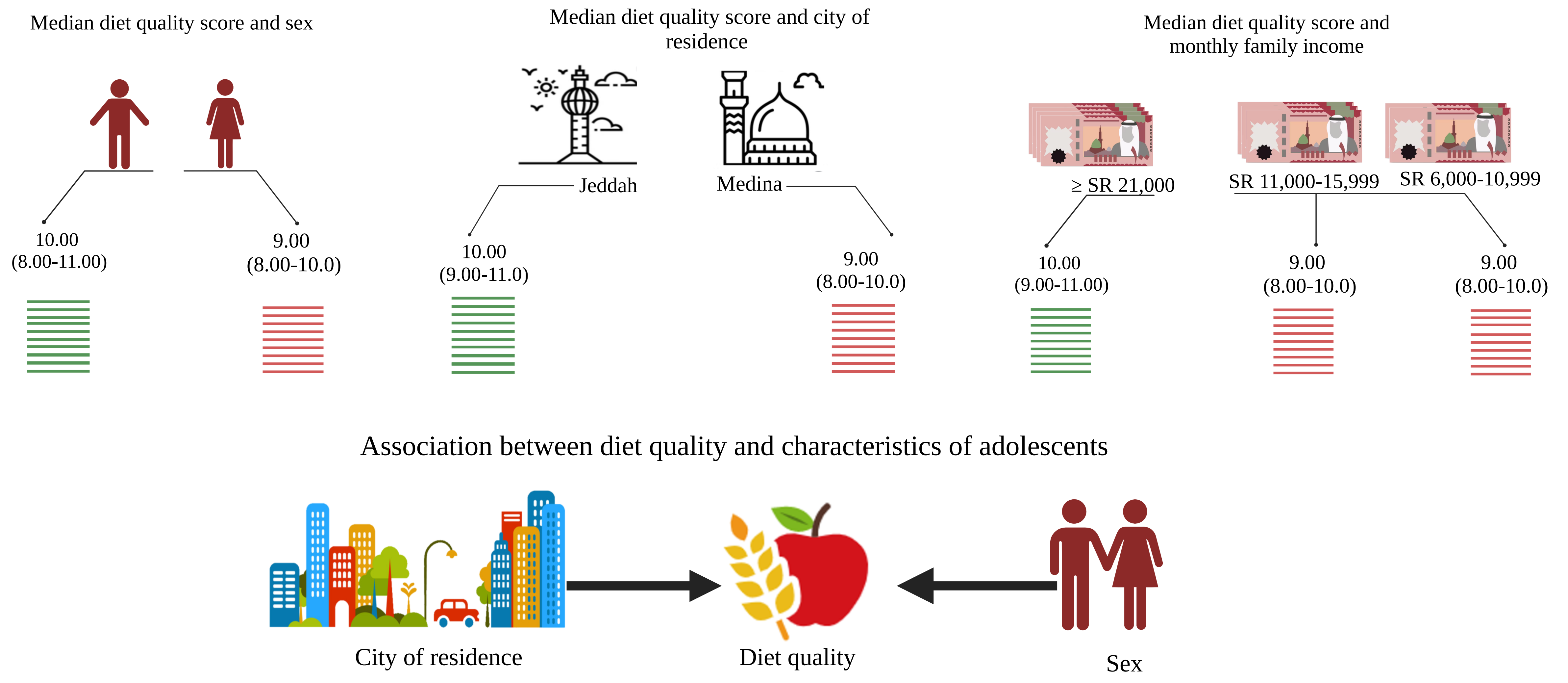

Supplement: Supplementary file 1 [file Data_Sheet_1.PDF]
